# Editorial: Pathophysiological and clinical insights for atrial fibrillation/flutter or heart failure

**DOI:** 10.3389/fcvm.2023.1221878

**Published:** 2023-07-25

**Authors:** Chun Lin, Jianfeng Liu

**Affiliations:** ^1^ Department of General Medicine and Geriatrics, Shenzhen Qianhai Shekou Free Trade Zone Hospital, Shenzhen, China; ^2^ Department of Cardiology, the Second Medical Center and National Clinical Research Center for Geriatric Diseases, Chinese People's Liberation Army General Hospital, Beijing, China

**Keywords:** atrial fibrillation, atrial flutter, heart failure, pathophysiologic, clinical insights

**Editorial on the Research Topic** Pathophysiological and clinical insights for atrial fibrillation/flutter or heart failure

Geriatric atrial fibrillation (AF) and atrial flutter (AFL) frequently coexist and complicate each other's treatment. Anti-arrhythmic drugs may not be effective, and cardioversion to sinus rhythm may be necessary. Anticoagulation therapy's side effects are also worthy of attention. Recent techniques, such as left atrial appendage occlusion, are becoming attractive for AF/AFL management. Novel drugs targeting specific ion channels are also being investigated. Despite progress in geriatric AF/AFL or heart failure (HF), their mechanisms and treatments require further investigation. The current research topic includes 13 studies on current advancements in mechanisms and treatment options for geriatric AF/AFL or HF.

## For HF

Elderly patients have a higher incidence of chronic heart failure (CHF), which can lead to acute kidney injury (AKI) and poor prognosis. In a retrospective cohort study, Hou et al. found that a decreased change in N-terminal pro-brain natriuretic peptides (NT-proBNP) may improve survival outcomes and prevent severe AKI in elderly patients with CHF. However, an excessive decrease in NT-proBNP can increase the risk of non-recovery of renal function following AKI.

In elderly patients with acute heart failure (AHF) and oliguria, high doses of loop diuretics are often ineffective and may adversely affect prognosis. Liu et al. conducted a retrospective cohort study and found that the addition of tolvaptan (TLV) effectively increased urine output and had favorable effects on alleviating AHF progression. TLV may also reduce the risk of all-cause mortality in elderly patients with AHF and oliguria.

## For AF/AFL

AF and AFL, these two “cousin” arrhythmias, might present relevant distinctions (Saglietto et al.). In particular, the ventricular response is completely irregular during AF, while generally regular during AFL, and their pathophysiology is different. The AF-related beat-to-beat variability alters deep cerebral microvascular perfusion as it induces transient and repetitive hypoperfusion or hypertension. A differential risk of dementia between AF and AFL was found in a national cohort study (1).

Research has shown that AF and AFL induce tachycardiomyopathy (TCM) which leads to reversible HF for many years. However, the knowledge of TCM is still limited. Ermert et al. demonstrated that approximately 5% of all patients hospitalized for HF suffer from AF/AFL-induced TCM. Age, NT-pro-BNP level, and resting heart rate >112 beats/minute can improve the discrimination between AF/AF- induced TCM and HFrEF with AF/AFL. These parameters may allow for an earlier diagnosis and improved therapy.

Atrial fibrosis represents a major hallmark in the disease progression of AF. Circulating microRNA-21 (miR-21) has been validated as a biomarker that reflects the extent of left atrial fibrosis in AF patients. Furthermore, Pradhan et al. revealed that under tachyarrhythmic conditions, miR-21-5p is released in-vitro from cardiomyocytes and stimulates collagen production by fibroblasts in a paracrine mode.

AF is prone to HF and stroke. Early management can effectively reduce the stroke rate and mortality. In a population of 18,738 elderly people (aged over 60 years old) in Chinese communities, He et al. found that a combination of age, atrial premature beats, atrial flutter, left ventricular hypertrophy, hypertension, and heart disease may be used to screen high-risk individuals for AF episodes.

According to Guo et al., elderly Chinese patients with AF are generally multimorbid and polypharmacy. They also report a high rate of inappropriate prescribing. The ABC (Age, Biomarkers, and Clinical history)-bleeding risk score may be useful in assessing the risk of major bleeding in Chinese patients with AF on oral anticoagulation therapy in real-world practice, and they suggest that this score performed better in stratifying patients with a high risk than the modified HAS-BLED score (Wang et al.).

The angiotensin receptor-neprilysin inhibitor (ARNI) is a potential upstream treatment option for AF. ARNI could reduce atrial electrical instability in AF in comparison with ARB in both retrospective studies and animal experiments (Zhu et al.).

The left atrial appendage (LAA) and structure may be predictors of AF recurrence after CA, but the results of studies evaluating them are contradictory. A meta-analysis (Han et al.) showed that AF recurrence after CA is more likely in patients with large LAA structures (LAA volume, orifice area, orifice long/short axis, and volume index) and decreased LAA function prior to ablation (LAA emptying flow velocity, filling flow velocity, ejection fraction, and LASEC).

Atrial fibrosis is associated with left atrial low-voltage areas (LVAs); however, it is unclear how these areas affect recurrence after CA. According to Mao et al., LVAs can reduce the risk of arrhythmia recurrence after conventional ablation in AF patients. Moreover, additional substrate modification in LVAs patients could reduce the possibility of arrhythmia recurrence.

AF-CA is associated with a number of serious complications, including acute pericardial tamponade (APT). In pericardial autotransfusion (DAT), pericardial blood is injected directly into patients' veins without a cell-salvage system. There is limited information available regarding DAT for APT. Zhao et al. displayed that DAT could be a feasible and safe method to deal with APT during the AF-CA procedure.

Inflammatory conditions such as periodontitis (PD), a chronic inflammatory disease, may contribute to the development of AF. According to Tashiro et al., PD is independently associated with an increased risk of arrhythmia recurrence after the first CA for paroxysmal atrial fibrillation.

As a whole, the current Research Topic provides up-to-the-minute information on many aspects of pathophysiology and clinical considerations associated with AF/AFL or HF. New perspectives may emerge as a result of more comprehensive knowledge based on these discoveries.
